# Oral acyclovir induced hypokalemia and acute tubular necrosis a case report

**DOI:** 10.1186/s12882-018-1121-0

**Published:** 2018-11-14

**Authors:** Jonathan S. Chávez-Iñiguez, Ramón Medina-Gonzalez, Lilia Aguilar-Parra, Eduardo J. Torres-Vázquez, Pablo Maggiani-Aguilera, Enrique Cervantes-Pérez, Guillermo García-García

**Affiliations:** 1 Servicio de Nefrología, Hospital Civil de Guadalajara Fray Antonio Alcalde, Guadalajara, Mexico; 2 Centro Universitario de Ciencias de la Salud CUCS, Universidad de Guadalajara, Guadalajara, Mexico; 3 Servicio de Medicina Interna, Hospital General de Occidente, Guadalajara, Mexico

**Keywords:** AKI, Acyclovir, Hypokalemia

## Abstract

**Background:**

Acyclovir is one of the most common prescribed antiviral drugs. Acyclovir nephrotoxicity occurs in approximately 12–48% of cases. It can present in clinical practice as acute kidney injury (AKI), crystal-induced nephropathy, acute tubulointerstitial nephritis, and rarely, as tubular dysfunction. Electrolytes abnormalities like hypokalemia, were previously described only when given intravenously.

**Case presentation:**

A 54 year-old female presented with weakness and lower extremities paresis, nausea and vomiting after receiving oral acyclovir. Physical examination disclosed a decrease in the patellar osteotendinous reflexes (++ / ++++). Laboratory data showed a serum creatinine level of 2.1 mg/dL; serum potassium 2.1 mmol/L. Kidney biopsy was obtained; histological findings were consistent with acute tubular necrosis and acute tubulointerstitial nephritis. The patient was advised to stop the medications and to start with oral and intravenous potassium supplement, symptoms improved and continued until serum potassium levels were > 3.5 meq/L.

**Conclusions:**

The case reported in this vignette is unique since it is the first one to describe hypokalemia associated to acute tubular necrosis induced by oral acyclovir.

## Background

Drug-induced kidney injury is a frequent adverse effect seen in clinical practice by different mechanisms that commonly lead to acute kidney injury (AKI) [1]. In a large cohort study of patients with community-acquired AKI (CA-AKI), nephrotoxic drugs where assumed in 59.9% of the cases [2].

Adverse drug reactions (ADRs) have a major impact on public health. Between 1999 and 2008, the annual number of ADRs increased 76.8%, and a two-fold increase in drug nephrotoxicity was observed [3].

Antiviral drugs like acyclovir, are known to induce AKI when administrated intravenously. Risk factors for developing acyclovir-induced nephrotoxicity include hypovolemia, rapid intravenous infusion, concurrent AKI prior to drug administration, excess medication dosage in relation to renal function, and concurrent use of other nephrotoxic agents [4]. Severe nephrotoxicity occurs in approximately 12–48% of the cases [5]. However, the potential damage induced by oral administration remains unclear. In one study aimed to assess hospital admission due to AKI within 30 days after prescription of oral acyclovir, it was found that neither the use of acyclovir nor valacyclovir was associated with a higher risk of AKI, in comparison to famcyclovir [6, 7].

Potassium disturbances are a well-known harmful consequence of drug intake. Drug-induced hypokalemia is most frequently caused by diuretic [8] glucocorticoid [9], and laxative administration [10].

Alternatively, it may be the result of an increased intracellular potassium influx induced by the use of sympathomimetic drugs [11], and insulin [12].

We report a unique case of oral Acyclovir induced hypokalemia and CA-AKI due to acute tubular necrosis.

## Case presentation

### Clinical history and laboratory data

A 54 year-old female presented to the outpatient clinic with weakness and lower extremities paresis, nausea and three times vomiting, after receiving clindamycin, dicloxaciline, and oral acyclovir 400 mg each 8 h were prescribed to treat a dental abscess. Relevant past medical history included allergy to penicillin and smoking for 20 years. Physical examination disclosed good hydration status, decrease in the patellar osteotendinous reflexes (++ / ++++). Initial laboratory data showed a serum creatinine level of 2.1 mg/dL; blood urea nitrogen 86.4 mg/dL; serum potassium 2.1 mmol/L, sodium 134 mmol/L, phosphorus 1.7 mg/dL, and magnesium 2.15 mg/dL. (Table 1).

**Table 1:** Laboratory data

Parameters	Baseline	Day 3	Day 11	Day 13	Day 14	Day 15	Reference range
Hemoglobin (g/dL)	13.3	12.8	12.5	10.82	11.1	-	12.0–16.0
Hematocrit, %	35.4	33.8	36.7	31.8	32.45	–	37–47
Platelets, K/mcl	326,000	318,000	253,700	207,100	203,600	–	150,000 - 500,000
White blood cells (K/mcl)	6290	5790	4780	3860	4120	–	3600–11,600
Glucose (mg/dL)	136	123	100	159	139	–	60–99
Urea (mg/dL)	86.4	76.3	63.8	45.8	42	–	16.6–48.5
Creatinine (mg/dL)	2.1	1.7	1.18	1.1	1.14	–	0.5–1.2
Uric acid (mg/dL)	8.1	7.3	5.4	4.8	5	–	2.4–5.7
Potassium (mmol/L)	2.1	2.5	3	2.8	2.8	3.1	3.5–5.1
Sodium (mmol/L)	134	139	140	140	141	139	135–145
Calcium (mmol/L)	10.1	9.7	9.3	8.5	8.5	8.9	8.6–10
Cloride (mmol/L)	89	95	106	108	109	102	98–107
Phosphorus (mg/dL)	1.7	2	2.9	3	3.4	2.2	2.5–4.5
Magnesium (mg/dL)	2.15	1.88	–	–	–	–	1.6–2.6
Albumin (g/dL)	4.9	4.8	4.1	3.3	3.5	–	3.5–5.2
Blood pH	–	7.45	–	–	–	–	7.35–7.45
CO_2_ mmHg	–	37.2	–	–	–	–	35–45
HCO_3_ (mmol/L)	–	25.3	–	–	–	–	23–25
Urine pH (dipstick)	7	7	6.5	–	–	–	5.0–8.0
Dipstick protein, mg/dL	negative	negative	negative	–	–	–	Negative
Red blood cells (dipstick)	negative	negative	negative	–	–	–	Negative
leukocyte esterase (dipstick)	negative	negative	15	–	–	–	Negative
Urinary casts	none	none	None	–	–	–	None
Urine protein (g/day)	–	–	–	0.23	–	–	
Urine potassium (mmol/day)	–	49	–	86	–	–	< 30
Urine sodium (mmol/day)	–	86	–	162	–	–	
Urine calcium (mmol/day)	–	89	–	57	–	–	
Urine phosphorus (mg/day)	–	–	–	143	–	–	
Spot urine potassium (mEq/L)	–	20.1	–	30	–	–	< 15
Spot urine sodium (mEq/L)	–	37	–	57	–	–	< 20
Spot urine calcium (mEq/L)	–	–	–	1.73	–	–	
Spot urine phosphorus (mEq/L)	–	–	–	4.6	–	–	

### Additional investigations

The patient was advised to stop the medications and to start with oral potassium supplement. She noticed improvement of the weakness; however, due to persistent hypokalemia, the patient was admitted to the hospital for further evaluation; new blood test showed a serum creatinine level of 1.7 mg/dL and blood urea nitrogen 76.3 mg/dL; serum potassium 2.5 mmol/L, sodium 139 mmol/L, phosphorus 2 mg/dL, and magnesium 1.88 mg/dL; urine sediment was unremarkable; 24 h urine potassium was 49 mEq/day, sodium 86 mEq/day, and calcium 89 mg/day. A renal ultrasound showed a normal-size kidney, without hydronephrosis. Kidney biopsy was performed.

### Kidney biopsy

**Fig. 1:**
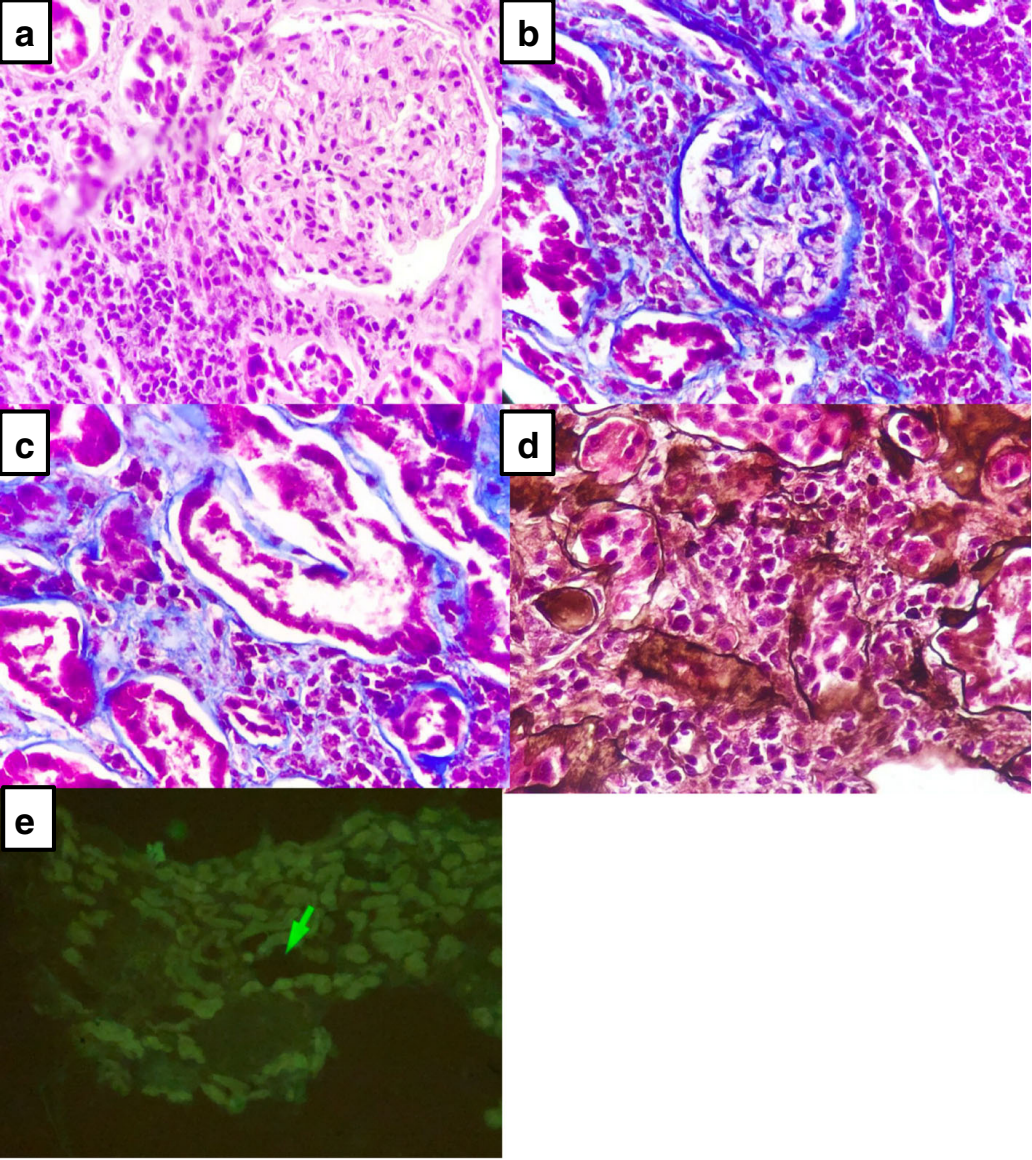
**a** and **b** Mesangium without proliferation and open capillaries, tubules with brush edge loss and cytoplasmic vacuolization, with diffuse inflammatory infiltrate. **d** Acute tubular necrosis, (**c**) tubulointerstitial nephritis with abundant inflammatory infiltrate and (**e**) immunofluorescence negative for immune complexes

Two fragments of kidney tissue were obtained (Fig. 1), By stereoscopic evaluation 11 glomeruli were identified. By light microscopy 9 glomeruli were observed; three were globally sclerosed and the remaining glomeruli were normal. Tubular atrophy was seen in 15–20% of the tubules; the remaining tubules showed vacuolated, granular cytoplasm, focal sloughing of the epithelium, and regenerative changes of the brush edge of tubular cells and hyaline casts (Fig. 1a). By Masson’s trichrome stain (Fig. 1b and c), the interstitium exhibited 0–15% fibrosis, with inflammatory infiltrate by lymphocytes, plasma cells, and few eosinophils, that penetrated the tubular epithelium. Arterioles and medium-caliber arteries were permeable without vasculitis or thrombi. By Jones (Fig. 1d) tubule-interstitial nephritis infiltrated with abundant inflammatory cells were observed. Immunofluorescence (Fig. 1e) was negative for IgM, IgG, IgA, C3c, C1q, kappa, lambda, fibrinogen, and albumin. Histological findings were consistent with acute tubular necrosis and acute tubulointerstitial nephritis.

### Clinical follow-up

Due to persistent hypokalemia oral and intravenous potassium replacement was started with good tolerability and no adverse events, symptoms improved; the patient was discharged 4 days later with a serum potassium of 3.1 mEq/L; oral replacement of potassium was continued until serum potassium levels were > 3.5 meq/L, after few days later she restart her normal life.

### Diagnosis

Hypokalemia and acute kidney injury due to oral acyclovir.

## Discussion and Conclusions

Ramírez et al. have described acyclovir and other drug-induce causes of hypokalemia. Drug-induced severe potassium disturbances were found in 32.3% of the cases, and in 23% were lethal; hypokalemia was present in 23.4% of life-threatening potassium disturbances [7].

Intravenous acyclovir is the most common therapy against herpes virus; it can induce crystalluria and lead to AKI. In a population-based study, hypokalemia developed in 46% of patients receiving iv acyclovir compared to 21% in the control group; individuals with a previous normal potassium levels, altered estimated glomerular filtration rate, or previous use of diuretics were at higher risk of developing hypokalemia [13]. They suggested that iv acyclovir induces hypokalemia by reduced renal plasma flow and/or by crystal-induced distal tubular damage.

**Fig. 2:**
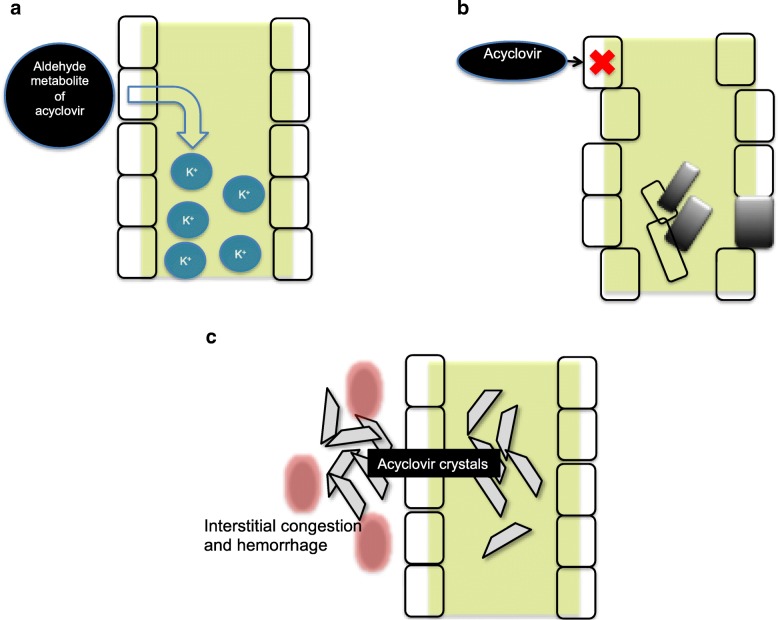
Effects of acyclovir on the renal parenchyma. **a** Tubular dysfunction and electolyte loss; **b** Direct injury to the renal tubular cell, necrosis and tubular obstruction; **c** Crystalluria and crystals in the tubular parenchyma

Other possible mechanisms include tubular dysfunction mediated by aldheyde metabolite of acyclovir; this complication could explain the urinary loss of potassium and others electrolytes (Fig. 2a); by direct injury to the renal tubular epithelia causing tubular cell degeneration and sloughing. Indeed, renal biopsy findings of patients with acyclovir toxicity include bulging of tubular cells, dilated tubular lumens, loss of proximal-distal tubular differentiation, flattening and vacuolization of epithelial cells, and epithelial cells mitoses [5] (Fig. 2b); and crystalluria**,** which usually develops 24–48 h after the initiation of acyclovir therapy due to its low solubility in urine. Severe intraparenchymal crystal precipitation can cause interstitial congestion and hemorrhage, leading to a decrease of renal blood flow (Fig. 2c) [14].

Acyclovir does not seem to affect the tubular handling of creatinine, suggesting that the pronounced, transient elevation in plasma creatinine, may be due solely to decreased glomerular filtration rate (GFR) as a result of renal dysfunction [5].

To prevent the possible the adverse effects of acyclovir, it has been recommended to establish euvolemia, slow intravenous infusion (over 1–2 h), adjusting the dose according to renal function, and to avoid the use of concomitant nephrotoxic agents prior to acyclovir administration [4].

It is important to mention that a great limitation of the presentation of our case is that we cannot adjudge causality for the intake of acyclovir, but there is biological and clinical plausibility that sustains this event. Strength of our case is that we have obtained renal tissue for histological analysis, since it is not common to perform a kidney biopsy in the presence of acute kidney Injury secondary to drugs.

The detection, reporting, and prevention of ADRs due to acyclovir, are essential to provide appropriate, safe, and effective therapy with this drug.

Acyclovir nephrotoxicity occurs in approximately 12–48% of cases, is associated with 3 patterns of kidney injury: tubular dysfunction and electrolyte loss, direct injury to the renal tubular cells and crystalluria, in 46% of patient that recive IV acyclovir develop hypokalemia. Probably both IV and oral acyclovir are associated with hypokalemia. To prevent the possible the adverse effects of acyclovir, establish euvolemia, slow intravenous infusion, adjusting the dose to renal function, avoid concomitant nephrotoxic agents. Early detection of renal and tubular dysfunction of ADRs due to acyclovir, are essential to provide appropriate, safe, and effective therapy with this drug.

## Abbreviations

ADRs: Adverse drug reactions
AKI: Acute kidney injury
CA-AKI: Community-acquired acute kidney injury
GFR: Glomerular filtration rate
IV: Intravenous
